# Endometriosis: patient–doctor communication and psychological counselling

**DOI:** 10.1007/s00404-023-07292-2

**Published:** 2023-12-05

**Authors:** Tewes Wischmann, Beate Ditzen

**Affiliations:** 1 Institute of Medical Psychology, Heidelberg University Hospital, Bergheimer Str. 20, 69115 Heidelberg, Germany; 2 University of Heidelberg, Heidelberg, Germany

**Keywords:** Endometriosis, Psychosomatics, Patient–doctor communication, Trauma, Infertility, Couple relations, Sexuality

## Abstract

The fact that endometriosis is one of the most frequent gynaecological disorders in women and girls is gradually attracting greater public and political attention. There is also substantial awareness of the disorder among German gynaecologists, albeit without sufficient “equipment” for discussing the condition with patients and providing psychological counselling. This article offers hands-on proposals for medical counselling and the discussion of relevant issues, as well as other practical tips and suggested verbalisations for use by women suffering from endometriosis (and their partners). These practical recommendations will certainly help to improve the doctor–patient relationship in the case of endometriosis. The resources mentioned below (guidebooks, websites) focus on German-language offerings only.

## What does this study add to the clinical work

**Table:** 

Endometriosis patients may experience themselves as passive/dependent and helpless in the face of the disorder. With a view to enhancing the quality of life, they should thus be encouraged to engage in active/self-reliant self-management. This paper shows example interventions in medical counselling that help empowering endometriosis patients.	

## Introduction

Endometriosis is a disorder in which tissue similar to the lining of the uterus grows outside the uterus itself. Depending on female sex-hormone fluctuation, the tissue grows and contracts much like the lining, thus frequently causing pain at the points where it is located. Where endometriosis makes an appearance, immune response may also be affected, producing an adverse effect on fertility. Endometriosis is a chronic disease frequently causing severe pain during menstruation, sexual intercourse, defecation and/or urination, chronic pelvic pain, bloating, nausea, fatigue and occasionally depression and anxiety. Globally, some 10% of women and girls at child-bearing age (190 million) are affected by endometriosis, in patients undergoing fertility treatment the percentage is anything up to 30% [1]. At present, endometriosis can only be reliably diagnosed by means of laparoscopy. This is one reason why a very long time may elapse (7 years on average) between initial symptom perception and diagnosis. In addition, the variability and the wide range of symptoms make it difficult for health care workers to diagnose endometriosis, while many women suffering from the disorder know little or nothing about it. A misinterpretation of the symptoms as “psychological” may have an adverse effect on patient–doctor relations [2, 3]. This refers in particular to the patient–doctor communication regarding the pain caused by endometriosis and its experience [4].

As there is at present, no known cure for endometriosis, treatment normally aims at controlling and ameliorating the symptoms. Access to early diagnosis and effective treatment is important but in many cases restricted, notably in countries with low- or medium-income rates. Globally, more research and greater sensitisation is needed to enhance prospects of effective prevention, early diagnosis and better management of the disease [5].

## Psychic aetiopathogenetic factors

There have been various conjectures forwarded to explain the psychic aetiopathogenesis of endometriosis. Some outdated accounts from the field of psychosomatic gynaecology interpret endometriosis as an expression of unresolved unconscious conflicts, one of them speculating that the disorder reflected a rejection of men on the part of these patients [2]. In a dissertation dating from 1975, we find the following verdict: “Ongoing dysmenorrhoea can be classified (…) as an expression of aggressiveness towards men” (op. cit., p. 233), a conclusion cited uncritically as late as 2009 [6, S. 453]. Similar blanket judgments of a pathologising nature can be found in an overview of 2003: “rejection of maternity and gender roles”, “unresolved conflicts associated with menstruation”, “disturbed sexuality and partnership problems”, “marked auto-aggressive potential”, etc. [7]. Some recent guidebooks also contain ascriptions of this kind: “Interestingly, I found mother conflicts in almost all the anamneses of endometriosis patients […]. Once the mother conflict is resolved, the endometriosis may gradually disappear of its own accord” [8, S. 100f]. In the face of such undifferentiated statements devoid of scientific evidence, we can only concur with Mary Lou Ballweg, the former president of the Endometriosis Association, when she refers to this as a case of “blaming the victim” [s. 2, S. 233].

Further speculation in conjunction with endometriosis revolves around the question whether in childhood or adolescence the women affected were exposed to sexual abuse and/or emotional neglect and whether this can be rated as a (part-)causal factor in the emergence of the disorder. In a study from 2006, 51% of the endometriosis patients questioned indicated that they had suffered sexual abuse in childhood or adolescence [over and against 34% in the control group without pain symptoms, cf. 2, p. 233]. A more recent study of 421 endometriosis patients and an equally large control group produced an odds ratio of 1.1 for sexual abuse (marginally significant statistically) and 1.2 both for emotional abuse and a combination of the two over and against the control group [9]. The study by Netzl et al. compared two equally large groups of endometriosis patients, one reporting severe gynaecological pain, the other none or little. In the first group, the investigators found a prevalence of 49% for reported traumatic events (as opposed to 32% in the comparison group). However, the difference was not statistically significant [10]. A more recent French study comparing endometriosis patients and a control group with regard to sexual abuse in their prehistory found no difference between the two [11]; but in both groups, sexual traumatisation referred to in anamnesis was associated with an increased experience of pain (similar to [12]). This very recent study confirms that in the interpretation of surveys relating pain-associated conditions to traumatic experiences, we need to bear in mind that traumatic experiences will generally reduce pain tolerance and that persons affected by early-childhood and/or repeated traumas will be more likely to develop a pain disorder. Persistent pain may lead to a greater likelihood of diagnosing endometriosis in trauma-sufferers than in non-traumatised patients. One critical reservation vis-à-vis studies that are retrospective in nature is that self-reported “memories” may be used to achieve “interpretive control” over the origins of the disorder and thus find for oneself a plausible way of explaining them and hence attaining greater potential control over them [“recall bias”, s. 2, S. 233]. Naturally, these deliberations are in no way intended to play down the usually disastrous consequences of real sexual and/or emotional abuse or the significance of the alarmingly high figures involved, pertaining as they do to just under 1/3 of the relevant members of the respective control groups.

There are no scientific data that endometriosis is induced by psychic cause, but there is a correlation between pain and e.g. previous trauma. Therefore, interventions designed primarily for women with traumas can be useful in the communication with endometriosis patients too. Such interventions include the stabilizing imaginative exercises described by Ellen Spangenberg in her excellent advice book (exercises pertaining to a safe and secure inner location like the “inner garden” or the “strongbox exercise” or to sources of strength like the “tree exercise”) [13]. The “emergency suitcase” described elsewhere in the book can also be recommended to endometriosis patients as a resource in times of intensive stress and profound despair (op. cit., pp. 83ff). Targeted "SOS tips" related to endometriosis, examples of positive affirmations and other practical advice can be found in the highly recommended current guidebook for endometriosis patients by Vivian Vanessa Wagner [14].

## Psychic impact of the disorder

Endometriosis may be associated with psychic symptoms ranging from despair, overstrain, helplessness and fatigue [15] all the way up to anxiety syndromes and depressive symptoms. These psychic aspects of endometriosis can also be allotted to the different facets of a disease as set out in the Research Domain Criteria (RDoC) and used to evaluate holistic therapy approaches at these levels [16].

The multiple (potential) repercussions of endometriosis on partnerships, social environment and training/work contexts are discussed below.

Endometriosis patients may experience themselves as passive/dependent and helpless in the face of the disorder. With a view to enhancing the quality of life, they should thus be encouraged to engage in active/self-reliant self-management. This would involve an active search for reliable and well-founded information on endometriosis (e.g. at www.endometriose-vereinigung.de or in the AWMF or ESHRE guidelines). Applying to a certified endometriosis centre and attending meetings of self-help groups will enhance the patients’ self-efficacy. In contrast, internet forums for endometriosis patients cannot be wholeheartedly recommended. In some cases, the search algorithms of these forums give pride of place to counsels ranging from paramedical half-truths to tips that have no basis in reality and are sometimes downright harmful. In short, they provide little genuine assistance.

## Essential factors in communication on endometriosis

The psychic symptoms encountered in connection with endometriosis include recurrent menstrual pain, anxiety and depressive symptoms as well as concerns about all those topics potentially associated with chronic pain disorders or impaired fertility. Accordingly, doctor–patient communication, counselling or psychotherapy will make use of components from specific therapies and also draw upon more general approaches. The following examples and verbalisation aids illustrate the point.

The main pillars of patient–doctor communication in this disorder are validation, normalisation and de-pathologisation in conjunction with externalisation. *Validation* means first of all confirming the intensity of the patients’ perceptions of pain and not playing them down or devaluing them as simulation [17]. Validation should also extend to feelings of helplessness and futility because recognition of these feelings is the indispensable precondition for the subsequent acceptance of the condition without which this disorder can hardly ever be overcome. *Normalisation* and *de-pathologisation* serve to relate the symptoms of the disorder to the overall syndrome as a whole, comparing and contrasting them with reports from other endometriosis patients.
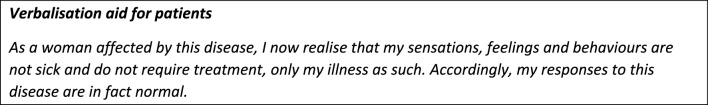


Here,* externalisation* (as a technique in systemic therapy) can mean distinguishing the illness from the personal identity of the individual affected with a view to achieving a new perspective on the disease by means of inner distancing. This can activate potential resources.
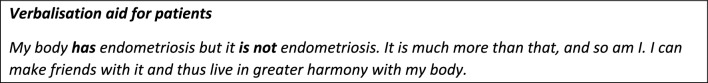


In this connection, the concept of “*posttraumatic growth*” has its place [18]. In communication with the patient, it is helpful to indicate that while in most cases the origin of endometriosis cannot be traced back to earlier traumas, the concept of posttraumatic growth still makes a great deal of sense in coming to terms with the illness. It expresses the fact that people can emerge from emotionally extremely distressing circumstances and from potentially traumatic events with greater strength and assurance, not just reduced or weakened. Endometriosis is frequently associated with chronic pain—and often with the unfulfilled desire for a child (see below)—thus generating feelings of powerlessness, futility and despair [19], so that the psychological concept of “posttraumatic growth” can also usefully be drawn upon in connection with endometriosis patients. At least three factors are positively associated with posttraumatic growth: (a) the presence of positive emotions, (b) social support and (c) an ability to find meaning in what one has been through [18]. The first two points can be addressed directly (see below under “sources of vitality” and partner relations). In contrast, meaningful coping will normally only evolve gradually in connection with ongoing reflection on the course of one’s own life.

## Coming to terms with pain

In endometriosis, *pain* is the symptom with the greatest psychic repercussions. Accordingly, the prime focus of doctor–patient communication will revolve around elements of pain therapy. Once again: crucial is the validation of the endometriosis patients’ perceptions of pain. The patient should be informed in detail about the various kinds of pain (somatic vs. visceral) and the interaction between nociceptive and nociplastic pains with protective postures and pelvic-floor dysfunction [20]. It is beneficial for the development of the illness to instil an awareness that in the long-term endometriosis can make women suffering from it more sensitive to pain (nociplasticity) and that pain therapy needs to be modified accordingly. The term “pain memory” and the behavioural rules deriving from it should be explained as simply as possible (e.g. www.schmerzedukation.de). If and when pain increases, the timely recourse to analgesics is essential instead of waiting until one “cannot stand it anymore”[20]. Patients can be recommended to keep a pain diary to help them distinguish between cycle-dependent and cycle-independent or stress-conditioned pains, to understand the differing degrees of intensity involved and identify potential influence factors.

Pain can also be exacerbated by inherited attitudes (“face up to pain and it’ll go away”). Psychologically sound doctor-patient communication will seek to identify, address and query such homilies.
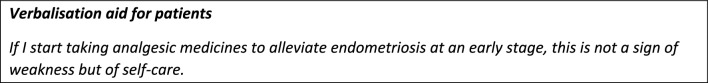


In addition, the entire range of pain-therapeutic measures (acupuncture, TENS, cannabis products, opioids, etc.) should be drawn upon, including systematic reference to pain specialists or pain centres. For everyday uses, warmth pads (in addition to hot-water bottles) can be recommended. Patients can also learn breathing techniques that midwives employ to alleviate labour pains. Protective postures adopted to alleviate pain are usually unphysiological. To prevent chronification, systematic pelvic-floor training with the support of physiotherapy specialists should be recommended.

One major potential source of pain exacerbation is *thinking the worst* [21]. There are highly successful therapeutic approaches, notably to chronic back pain, that proceed from this insight, engage with catastrophic thinking and give it a positive twist [22]. This approach can also be used in connection with chronic pelvic pain. Among the *imaginative exercises* that can help come to terms with pain, patients can be recommended exercises like the “light” or “waterfall” varieties [13, S. 141ff].

## Endometriosis therapy is multi-modal therapy

There is no causal therapy for endometriosis because the reasons for the disorder have yet to be fully elucidated. Accordingly, it will be advisable to understand the disease as a complex and probably multi-causal system, so that a combination of different approaches will be the option of choice in getting to grips with it.
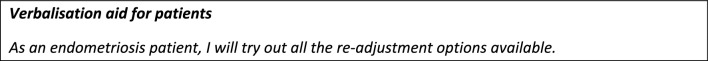


No single measure will work equally well for all endometriosis patients (for instance, there is no specific “endometriosis diet”). Accordingly, as with all chronic disorders, the promises of cure held out in books and on websites must be taken with a pinch of salt. Alongside hormonal (and surgical) therapy, pain-therapeutic and physiotherapeutic approaches have their legitimate place, as do dietary counselling, osteopathy, relaxation techniques, psycho-educative counselling and psychological chaperoning, plus psychotherapy and non-verbal therapy (dance, painting, art and music therapy).

## The internal attitude is crucial

Central elements operative in achieving a beneficial attitude to endometriosis are mindfulness and self-care, radical acceptance, delimitation, authenticity and self-efficacy.

In its original sense, *mindfulness* [23] stands for a non-evaluative attempt to observe one’s own perceptions, thoughts and feelings and to let them come and go without adhering to them. In connection with endometriosis, mindfulness means focusing on the here and now (“How exactly do specific parts of my body feel at this moment?”). But it also extends to an appreciation of the temporal dynamics of the complaints: “How did my body feel yesterday, what can I expect for tomorrow?” This combination of mindfulness and temporal structure can help patients desist from thinking the worst and release the psychic resources obstructed by such thoughts.
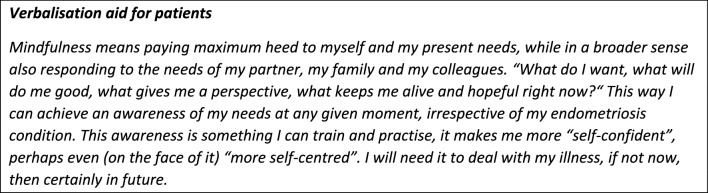


This leads on immediately to *self-care*.
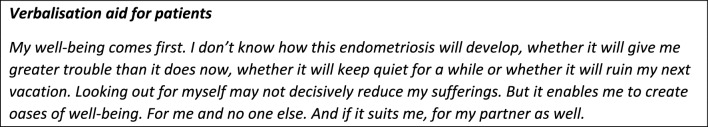


In this connection, the following questions from the doctor are helpful: “In what situations do you manage to forget endometriosis completely?” “Can you integrate those situations into your everyday life and possibly even extend them?” If the patient is having a hard time, e.g. if the pain gets worse or if there are serious conflicts at work or with her partner, self-care will involve always planning from one day to the next. It will be best not to make any over-ambitious plans that may come to nothing but to opt instead for realistic, small-scale “mini”-plans affording satisfaction and gratification when they turn out well. Big plans can always be broken down into smaller stages.

Psychotherapists dealing with chronic pain tell us that “radical acceptance” of the condition is a way of transferring the treatment effect to bodily functioning [24, 25].

*Radical acceptance* Dialectic behaviour therapy has come up with the concept of “radical acceptance” to refer to the acceptance of unchangeable emotions on the one hand and unchangeable circumstances on the other [26]:
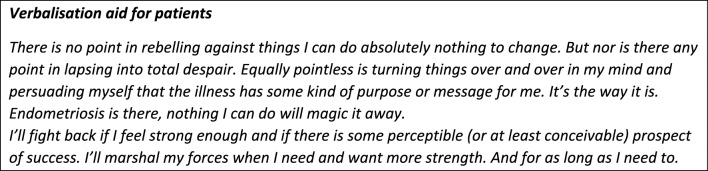


Recommendation for medical counselling: “Of course, radical acceptance does not mean denying the illness. On the contrary. If you acknowledge the limits imposed by endometriosis (or at least try to), then you can explore the options within and outside those limits. Nor does radical acceptance mean letting the illness drain you completely and then lapsing into resignation or depression.”

*Delimitation and authenticity* Every (chronic) disease will be easier to cope with if the affected persons concentrate on the essential tasks and challenges facing them in their present lives. Here, it is of course helpful to regularly practise mindfulness in order to focus on what is genuinely important for them at any given time. Mustering one’s strength may of course involve having to say “no” to others more often than usual and to actively reject claims that are overt, covert or possibly only imaginary. Such claims may only be present in the patient herself and may have long been internalised. But they may also be addressed to the patient from outside.
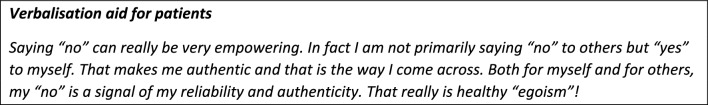


Like every chronic illness, endometriosis is often very largely bound up with feelings of helplessness. Permanent helplessness is a major risk factor involved in the onset of depression. An effective “buffer” against this is *self-efficacy* [27].
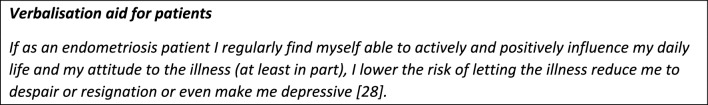


In the following, we propose a number of concrete and practical tips on how endometriosis patients can (re-)integrate the illness into their lives (instead of having to rebuild their lives around the illness with unsatisfactory results). What ultimately counts is getting enough enjoyment (back) into daily life to make it easier for the patient (and her partner) to deal with the illness.
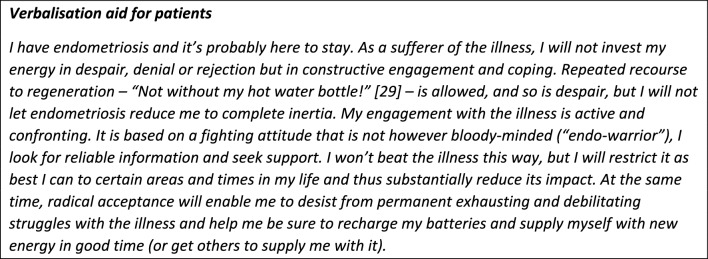


We know from coping research that active-avoidant *coping behaviour* can increase the negative psychological impact of a disease. In contrast, an active-confronting approach can reduce that impact. The same is true of meaningful coping strategies [30].

## Achieving a better body feeling

This should be a central concern for endometriosis patients.
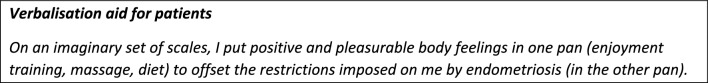


Various options can be recommended to patients for *improving their body feeling*. They include moderate endurance sport and individually adapted physical training, relaxation techniques (autogenic training, progressive muscle relaxation after Jacobsen, yoga, qigong, Tai-Chi, etc.), pelvic-floor training, water cures (including water gymnastics) and collective tissue massage. The options also extend to heat therapy, osteopathy, dance therapy, creative therapy (art and music therapy) and traditional Chinese medicine (TCM). Many of these physical activities/relaxation techniques and mindfulness exercises can also be learned by watching educational videos on serious internet portals (e.g. those of the health insurance institutions). Here again, the motto is: Try out all the re-adjustment options on offer and use the ones that do you good.

Recommendation for medical counselling: Patients with major *sleep disorders* (e.g. due to pain) should seek medical aid to improve their sleep quality. They should avoid self-medication with sleeping pills (possible interaction with pain-killers) and above all the use of benzodiazepines (addiction potential). The websites of the health insurance institutions and (say) www.psychologische-coronahilfe.de contain uncomplicated self-help tips designed to improve sleep quality.

Another major factor in achieving improved body feeling is *dietary counselling*. Many patients complain of the so-called “endo-belly”, i.e. cycle-dependent bloated belly [20]. Here, a change of diet can be helpful, e.g. a switch to a vegan diet devoid of gluten and histamines. So far, however, the only evidence-based indications we have are that that fish-oil capsules in conjunction with vitamin B12 help to reduce endometriosis symptoms and (largely) avoiding alcohol, red meat and trans fats has the same effect [31]. In communication with patients, it is also a good idea to point to the general recommendations for a balanced diet issued by the German Society for Nutrition and other institutions.

## Endometriosis and partner relations

One outcome that many—though not all—studies come up with is that endometriosis has a *negative effect on partner relations*. Ten percent of the participants in one international online survey indicated that the illness was one of the reasons for divorce [32]. One caveat in this connection is (probable) self-selection by the participants (people with problems are more likely to take part in online surveys about the effects of illness). We know from a recent online survey about the desires of endometriosis patients and their partners that in partner relations open, respectful and empathic communication from the outset is extremely beneficial [3]. Another desideratum was the enhanced awareness of the situation achievable via appropriate information for partners on all aspects of endometriosis, not merely the medical factors involved. Pro-active prevention strategies hindering the disease from gaining dominance over all aspects of the lives of both partners were also listed as a positive factor. This included freedom for both partners to create greater latitude for themselves without developing a guilty conscience vis-à-vis the respective other.

Studies on the partnership dyad all conclude that partners will have a major impact on the patient’s subjective experience and behaviour. This is the case, for example, in connection with coping strategies [30] and self-efficacy [28]. A study conducted in Heidelberg and Innsbruck indicated that the subjective experience of major stress heightened the negative impact of endometriosis pain not only on the patients, but also on their partners [33]. Interestingly, social support had a great deal less influence than the (subjectively perceived) absence of understanding for the illness from others. Here the (mutual) heightening of endometriosis pain was even more marked than in the subjective experience of stress. The conclusion is that it is imperative to extend the offer of psychologically sound counselling to the partners of endometriosis patients [34] and that therapy outcomes can be improved if therapy is addressed to both partners [35, 36]. The studies conducted so far have focused exclusively on heterosexual couples, but their findings will also be very largely applicable to lesbian relationships. As we have seen, it is very important to provide appropriate *information material* on endometriosis (and notably its psychological consequences) both for partners and for the general public (and hence for family members and colleagues).

## Strengthening resources: sources of vitality, energy bars and fortune-cookie inserts

Recommendation for medical counselling: “A chronic disease like endometriosis can periodically leave you drained and can attack your resources and powers. So it makes good sense first of all to perceive and identify your individual resources, actively foster and strengthen them and introduce exercises and rituals with which you can repeatedly replenish your *sources of vitality*. The options are legion. Start keeping a “good news” or “enjoyment” diary. Write your entries at the same time every day (e.g. shortly before retiring for the night). Identify four positive things that made your day more congenial. Ask yourself these four questions every day: ‘What was the good news today?’ ‘What did I do really well today?’ ‘What am I grateful for?’ ‘When did I feel really alive today?’” (cf. e.g. [18, S. 29] and [13, S. 88].

Another example of strengthening resources is creating “*energy bars*” (this is a recommendation for counselling with both partners present): “If you feel drained and exhausted, ask yourself what might recharge your batteries—and act on your own advice! Here are some suggestions: physical exercise in natural surroundings, regular sport, massage, music, dancing, meditation and yoga, meaningful conversations, sex and physical tenderness, good food, enough sleep, … Add energy bars of your own to the list.” “Both of you compile a personal list of 10 things that do you good. Stick up both lists (one for each partner) on the wall in a regularly frequented but private place (e.g. in the bathroom). Indulge in at least one of the 10 rules every day” [37, S. 29].

A glass jar for “*fortune-cookie inserts*” (a further recommendation for medical counselling). One participant in an internet infertility forum posted the following helpful idea for a “plan B”: “We also have a plan B. From now on we intend once a week to write down on a scrap of paper something that we either enjoy (e.g. a certain meal), or like to do (e.g. city trips) or want to do in future (e.g. a safari) and put it in a glass jar. This collection of “fortune-cookie inserts” is a constant reminder of all the good things in our lives, the things we should be grateful for and things we can look forward to” [38]. A (modified) strategy of this kind can be used in endometriosis counselling: “Imagine that endometriosis is really getting one or both of you down and you feel exhausted, desperate or helpless. Taking out a fortune-cookie insert can divert your attention from the intensely annoying restrictions imposed by the illness, remind you of the nicer things in life and point up new perspectives for the future”.

## Sexuality is much more than vaginal intercourse

*Sexuality* is central to intimate relations and also an important resource for improving body feeling. Here endometriosis can frequently play a highly disruptive role.
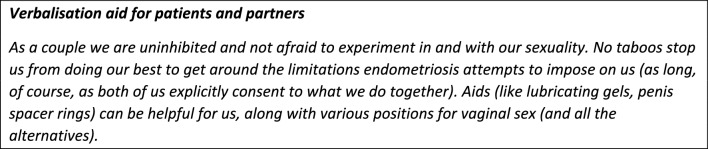


Pain during sex is an absolute “no go”. Unless there is explicit consent to the contrary from all partners involved, pain quite definitely has no place in sexual intercourse. When sexual practices approach the pain limit, it is time to desist from pursuing these practices (indicated by a sign or gesture agreed upon beforehand) and switch to gentler stimulation practices indulged in jointly on earlier occasions.

Recommendation for medical counselling: “Don’t let yourself be discouraged by negative experiences in your sex life you both have been through. Some feelings of pain that endometriosis patients suffer during intercourse are cycle-dependent, others are caused by stress (conventional or relational), others are (initially) inexplicable. Don’t be discouraged, keep trying! A new day means a new encounter with your partner and new prospects of fulfilment.”

The brochure “Endometriose, Sexualität, Partnerschaft” issued by the Endometriosis Network (www.endo-netz.org) contains valuable practical advice and suggestions on these issues and is accordingly a firm recommendation for patients and their partners.

## Endometriosis, social environment, effects on work and vocational training

At some point or other, every endometriosis patient has been forced by the illness to cancel get-togethers with friends. Accordingly, the patient should not mince her words but enlighten her environment about the disease in the following forthright terms:
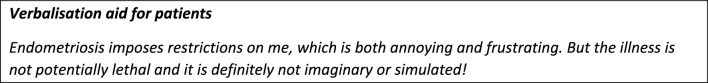


A frank and straightforward attitude to endometriosis (this is an aspect of the authenticity we discussed earlier) makes for dependability and predictability vis-à-vis other people (although it does nothing to make the illness itself easier to predict).

Like every chronic illness, endometriosis can also involve *social isolation*. The symptoms are unpredictable and inconsistent, which makes dealings with the patient uncertain and hard to plan, potentially causing impatience and exasperation. Frequently, endometriosis patients will plan (and call off) their dates with others on the basis of the menstruation cycle (which itself can however be anything but predictable). Some of the joint activities envisaged can be transferred to social media or video conferencing, but by no means all. Accordingly, not only partner relations are exposed to serious strain but also friendships (and family relations). We have seen earlier how important understanding on the part of others can be for the well-being of endometriosis patients (and their partners) [33].

In some cases, *vocational training* and *work* schedules can be affected by the illness. Here too, the best course is to adopt a pro-active and outspoken attitude to the condition and to inform employers accordingly. As far as is possible in the context of work or vocational training, patients should pro-actively and frankly bring up the subject of cycle-dependent (predictable) and cycle-independent (unexpected) pain episodes and the attendant eventuality of missed training sessions or absence from work. The situation can be facilitated by such things as a height-adjustable desk, flexible breaks, home schooling/home office. The international online survey referred to earlier established that endometriosis impaired half of the participants in their working capacity, while just about a quarter indicated that they had reduced their working hours accordingly [32].

Some rehabilitation clinics that provide care for patients with endometriosis have been certified by the Endometriosis Research Foundation (SEF) (www.endometriose-sef.de). Rehabilitation of this kind can be applied for as follow-on treatment (after inpatient care) or as a rehabilitation measure in its own right. Endometriosis patients have mostly judged these offerings to be very helpful and extremely professional. Patients with repeated massive restrictions in the work context should contemplate applying for an official ID card conferring disability status on them.

## Infertility and endometriosis: a special challenge

Endometriosis patients with an *unfulfilled desire for a child* usually suffer more acutely than others. Pregnancy would probably alleviate (but *not* cure) the illness, medication against endometriosis (hormone suppression) will delay pregnancy, about 2/3 of all patients suffer from pain during vaginal intercourse and endometriosis patients are frequently dependent on assisted reproductive technologies (e.g. because the fallopian tubes are affected by focal sites of the illness).

Valuable advice on this constellation can be found in the books by Anna Adamyan (née Wilken). She is an endometriosis patient with the desire for a child and in her two guides describes frankly and impressively what she has been through [39, 40]. Other examples of up-to-date and well-substantiated literature on involuntary infertility are [41] and [42]. The relevant AWMF guideline provides a good overview of evidence-based research findings on the subject [43]. In the case of involuntary infertility, psychosocial counselling experts can be found and contacted at www.bkid.de.

## Endometriosis patients and their gynaecologists

Endometriosis has only gradually established itself in the focus of public interest. Many women with the disease had experienced little sympathy for their complaints: “It can’t be so bad. Period pains are not an illness.” Formerly, this attitude was frequently voiced not only by mothers and girlfriends or colleagues but also by medical specialists (especially vis-à-vis younger patients) [44]. In her essay *Clinical Waste*, novelist Hilary Mantel gives us a graphic description of the situation: “The doctors told women who complained about painful periods that childbirth would sort them out. They told women who complained about pain throughout the month that they were hypochondriacs and nuisances, that they were really complaining about their bad marriages, that they could have a nice Valium if they liked and take it on a repeat prescription so you’re not back here every five minutes with the same old story.” [45, S. 20]. In the meantime, it is safe to assume that the *level of knowledge* about endometriosis *among gynaecologists* has greatly increased and with it the awareness of the specific problems involved. Ongoing training courses focusing on the illness support this development process, such as those offered by the AG Endometriose (www.ag-endometriose.de).

The international online survey referred to earlier concluded that the average period between symptom perception by the patient and correct diagnosis was 5.5 years [32]. This period is made up of an initially defensive attitude on the part of the patients lasting 2.1 years on average (“patients’ delay”) and a reluctance to commit themselves on the part of gynaecologists lasting 3.4 years on average (“doctors’ delay”). In practice, this indicates that women should not regard unusual and highly uncongenial symptoms (especially severe cycle-dependent pain) as something they “have to live with” but should insist on swift clarification by their doctors and an early transfer to a specialist endometriosis centre for diagnosis [46]. Naturally, this recommendation does not mean that every young woman with menstruation pains should be subjected to invasive endometriosis diagnostics (with imaging processes or even “routine laparoscopy”). In schools, however, more information should be imparted on the endometriosis syndrome [47]. The symptoms and basic diagnostic principles of endometriosis (diagnostic algorithm included) can be found in the latest AWMF guideline [48] and in the ESHRE guideline [49]. For patients and counselling experts, the medical aspects of the illness are described in straightforward language in Sylvia Mechsner’s guide to the illness [50].

## Conclusion

Endometriosis is an illness that for many patients (and their partners) involves a substantial impairment of everyday life and frequently takes a chronic course. Patient-oriented counselling can definitely profit from the principles of patient-doctor communication developed in connection with other chronic illnesses or traumas as they are equally applicable to endometriosis. Professional medical communication can (and should) clearly help to alleviate the (usually considerable) suffering associated with endometriosis.
